# The uncertainty of colombian scientific journals with Publindex

**DOI:** 10.25100/cm.v43i4.3789

**Published:** 2017-12-30

**Authors:** Mauricio Palacios

**Affiliations:** Editor en Jefe, Revista Colombia Médica. Universidad del Valle, Cali, Colombia

The colombian National Bibliographic Index, Publindex, has defined during the last two decades the management and editorial quality, the dissemination and even the number of scientific journals indexed in Colombia ^1^. The criteria with which Publindex accepts a scientific journal and qualifies it in this index are the road map of a large number of editorial committees and universities. These criteria have not changed much since their inception and corresponded to a minimum editorial approach (ISSN registration, creation of editorial committees, percentage of institutional endogamy, punctuality in publication, among others) rather than results in indicators of use and diffusion (indexation in specialized databases, citations, international collaboration, etc.). For the year 2010, the number of journals indexed in Publindex increased, and it slowed down the promotion of journals to higher categories ^2^.

Due to the above, Publindex adopted in that year a mixed classification system with four categories (A1, A2, B and C) based on the quartiles of the SJR (Scientific Journal Rankings) indexes of Scopus or JCR (Journal Citation Report) of Web of Science (normalized), and it superimposed the top two quartiles of the classification with the Google Scholar Metrics H-5 index (not normalized) in the two lower categories (B and C). With this system, in the convocation No. 768, Publindex recognized 33 journals in the health area (medicine, nursing and pharmacy); 16 because they are indexed in Scopus (Colombia has only two health journals in Web of Science in quartiles inferior to those classified in Scopus) and 17 with the quartile system H-5 of Google Scholar Metrics (GSM). This measurement was aligned with international systems; but it did not correct the editorial stagnation suffered by scientific journals; and there will emerge others if corrections are not early made.

The first problem in the measurement was to match so many different metrics in the B and C categories of the classification. SJR differentially evaluates the citations of a high-impact journal compared to the times cited in a low-impact journal (standardization of the metric); while GSM attributes the same value to a citation in a high-impact journal with respect to a citation in a gray literature document (non-standard metric). Therefore, editorial stagnation is encouraged in journals not classified in Scopus or Web of Science. Staying in categories B and C is relatively easy with GSM, as it is very vulnerable to increase citations artificially, because it takes into account documents that are not scientific articles and that can be published more expeditiously than a scientific article, due to the editorial process of a journal ^3^. 

**Figure 1:**
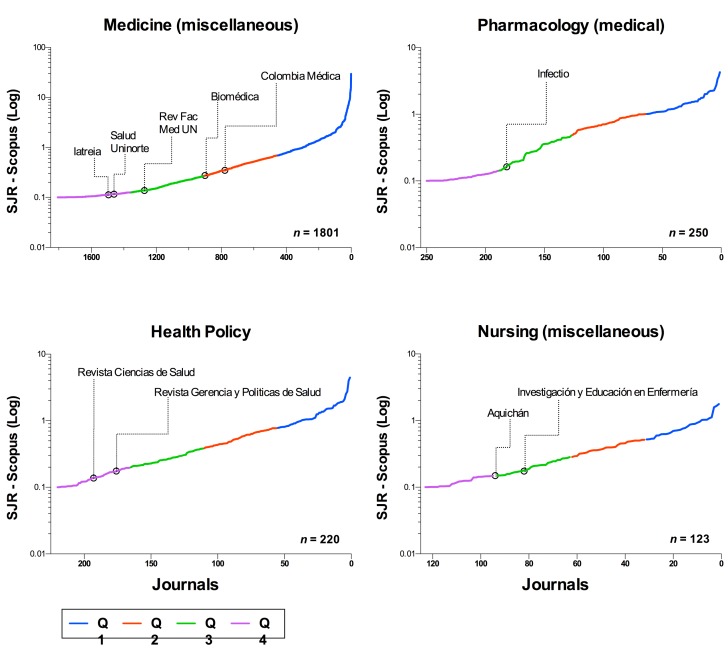
SJR rating of Colombian journals in four categories of the Scimago Journal ranking 2016. The promotion of a journal to have a better quartile rating requires, at least, duplicating the effort with each quartile because the requirements of the SJR measure have a logarithmic trend.

So far, the results of the normalization of data in Google Scholar with the "*Publish or Perish"* software are not satisfactory ^3^ ^,^ ^4^. This contrasts with the difficulty to ascend in the Scopus quartiles, which have a logarithmic tendency and the effort of a magazine to change the quartile is duplicated, regardless of the category (Fig. 1). Additionally, implementing citation strategies in GSM to maintain the Publindex category is unequal with respect to the effort to enter international citation databases and descend into the Publindex category (It would be expected that the journals with the best GSM rating, from category B, could aspire to Scopus; but they would be qualified as C when entering quartile 4). The pressure for immediate institutional results will give more weight to a decision to remain in the measurement with GSM, and the country will not grow with these indices.

**Figure 2:**
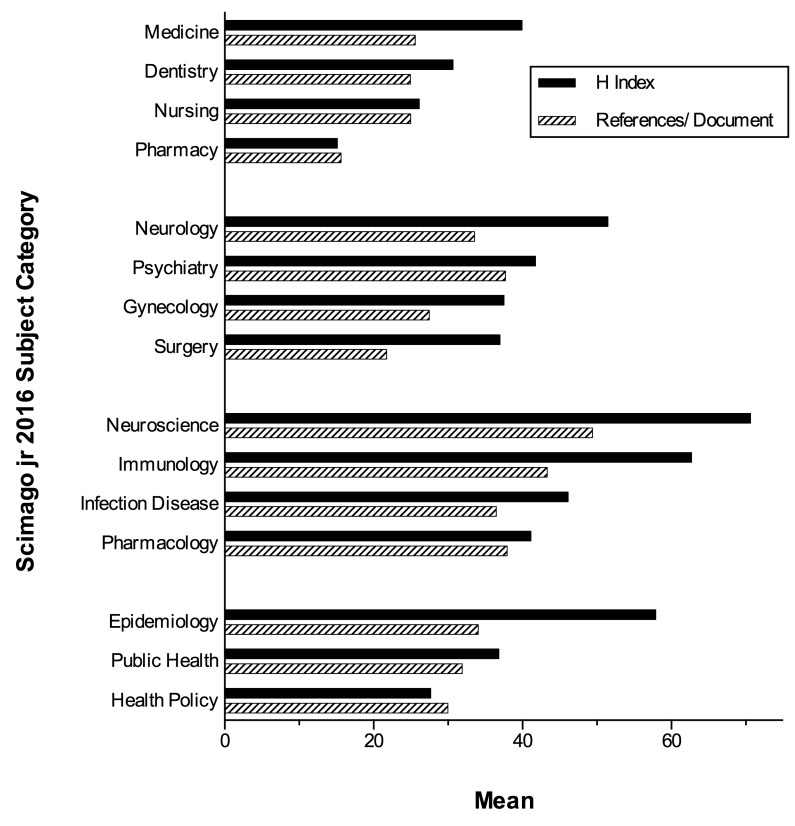
Production of articles and citations according to categories of the Scimago Journal Ranking in 2016. The variability in the citation habits (citations/document), and the citations received (represented by index H) mean that journals of different categories cannot be grouped together in the health area with Google Scholar Metrics.

Another problem is that bibliographic citation customs are different among the areas of social sciences compared with basic sciences and health areas. These differences are mainly noted in the number of citations and production of documents ^5^. This is also evident within the health area categories. Citations vary significantly among disciplinary categories such as medicine, nursing, dentistry, and pharmacy. The same happens with nearby categories such as neurology, psychiatry and neurosciences, or among clinical areas, basic sciences or public health categories (Fig. 2). For this reason, the grouping of magazines of different categories with GSM in large areas is inequitable.

With the above, it is easier to understand the underlying problems: the limited editorial development of the journals and the lack of knowledge of Colciencias to assume a measurement of the journals with the universal indexes. Using a single metric for national journals and approved international journals, coupled with the absence of training strategies and support for journals, will lead most Colombian journals to fragility. Brazil, a regional competitor, implemented with SciELO a powerful editorial content manager for its journals, Scholar One^TM^, improving the editorial processes and allowing an external audit in real time from SciELO ^6^ ^,^ ^7^. For a Latin American health journal, indexing in PubMed is more important than in citation bases; 90 have achieved it, among them, 51 Brazilians and 5 Colombians. But for Brazil, inclusion in PubMed Central is more important: 20 have been admitted in the last 5 years; and only one in the rest of Latin America: Colombia Médica. Citations and rankings come with the progress of the journal. If the singularity of each area is not understood, it is not possible to apply differences in the dynamics and the way of measuring the national magazines system. 

 Colombia is the third country in number of scientific journals in Latin America, which is why it is a regional reference in its models of production, edition, measurement and editorial projection. Publindex should reassess the measurement system proposed for the next convocations, defining the following characteristics: to maintain a mixed system, but not superimposed; with bets towards differential editorial development by areas and internationalization; to establish guidelines and subsidize solutions that improve the organizational, editorial and bibliometric deficiencies of the entire system; but that discourage the comfort of the magazines classified in the inferior quartiles. It is necessary the integration of the policies of other information systems in Colciencias, as the estimation of products in classification GrupLAC will strengthen the scientific journals in the medium term. But there remains the lack of promotion of peer evaluation, which is absent as a product in the categorization of CvLAC researchers; and the editors suffer the unquantified silence of the researchers better qualified by Colciencias when they are invited to evaluate the manuscript of a colleague. If the policies, strategies and convocations of Colciencias do not include and make visible values ​​such as equity, solidarity and reliability, it will be the main responsible for the delay of the investigation in this country.
