# Supplementary material for: The uncertainty of colombian scientific journals with Publindex
Source: Colomb Med (Cali). 2017 Dec 30;48(4):152–4. doi: 10.25100/cm.v43i4.3789 (PMC5896720; doi:10.25100/cm.v43i4.3789)
Supplement: Related texts

# Editorial: La incertidumbre de las revistas científicas colombianas con Publindex

El Índice Bibliográfico Nacional colombiano, Publindex, ha definido durante las últimas dos décadas la gestión y calidad editorial, la difusión y hasta el número de revistas científicas indexadas en Colombia ^1^. Los criterios con los cuales Publindex acepta una revista científica y la califica en este índice son la hoja de ruta de un gran número de comités editoriales y universidades. Estos criterios no han variado mucho desde sus comienzos y correspondían a un enfoque de mínimos editoriales (registro ISSN, conformación de comités editoriales, porcentaje de endogamia institucional, puntualidad en publicación, entre otros) más que a resultados en indicadores de uso y difusión (indexación en bases de datos especializadas, citaciones, colaboración internacional, etc). Para el año 2010, aumentó el número de revistas indexadas en Publindex y se desaceleró el ascenso de las revistas a las categorías superiores ^2^.

Debido a lo anterior, Publindex adoptó en este año un sistema de clasificación mixto con cuatro categorías (A1, A2, B y C) basado en los cuartiles de los índices SJR (*Scientific Journal Rankings*) de Scopus o JCR (*Journal Citation Report*) de Web of Science (normalizados), y le superpuso los dos cuartiles superiores de la clasificación con el índice H-5 de Google Scholar Metrics (GSM, no normalizado) en las dos categorías inferiores (B y C). Con este sistema, en la convocatoria N. 768, Publindex reconoció 33 revistas del área de salud (medicina, enfermería y farmacología); 16 por estar indexadas en Scopus (Colombia tiene solo dos revistas de salud en Web of Science en cuartiles inferiores a los clasificados en Scopus) y 17 con el sistema de cuartiles H-5 de Google Scholar Metrics (GSM). Esta medición se alineó con los sistemas internacionales; pero, no corrigió el estancamiento editorial que padecen las revistas científicas y emergerán otros, si no se hacen correcciones tempranamente. 

El primer problema en la medición fue equiparar métricas tan diferentes en las categorías B y C de la clasificación. SJR valora diferencialmente las citaciones de una revista de alto impacto respecto a las veces citadas en una revista de bajo impacto (normalización de la métrica); mientras; GSM le atribuye el mismo valor a una cita en una revista de alto impacto con respecto a una cita en una documento de literatura gris (métrica no normalizada). Por lo tanto, se estimula el estancamiento editorial en las revistas no clasificadas en Scopus o Web of Science. Mantenerse en las categorías B y C es relativamente fácil con GSM, pues es muy vulnerable a aumentar citas artificialmente, debido a que tiene en cuenta documentos que no son artículos científicos y que pueden publicarse de forma más expedita que un artículo científico, debido al proceso editorial de una revista ^3^. 

Hasta el momento, los resultados de la normalización de los datos en Google Scholar con la interface *Publish or Perish* no son satisfactorios ^3^^,^^4^. Esto contrasta con la dificultad para ascender en los cuartiles de Scopus, que tienen una tendencia logarítmica y el esfuerzo de una revista para cambiar de cuartil se duplica, independientemente de la categoría (Fig. 1). Adicionalmente, implementar estrategias de citación en GSM para mantener la categoría Publindex es desigual respecto al esfuerzo por ingresar a las bases de datos citacionales internacionales y descender en la categoría de Publindex (Se esperaría que las revistas con mejor calificación en GSM, de la categoría B podrían aspirar a Scopus; pero, serían calificadas como C al ingresar al cuartil 4). La presión por resultados inmediatos institucionales le dará más peso a una decisión de mantenerse en la medición con GSM, y el país no crecerá en estos índices.

**Figura 1:** Calificación SJR de revistas colombianas en cuatro de las categorías de Scimago Journal ranking 2016. El ascenso de una revista para tener una mejor calificación del cuartil requiere, al menos, duplicar el esfuerzo con cada cuartil porque los requisitos de la medida JCR tiene una tendencia logarítmica.

Otro problema, es que las costumbres de citación bibliográfica son diferentes entre las áreas de ciencias sociales con respecto a las áreas de las ciencias básicas y de salud. Estas diferencias se notan principalmente en el número de citas y de producción de documentos ^5^^).^ Esto, también es evidente dentro de las categorías del área de salud. Las citaciones varían significativamente entre categorías disciplinares como medicina, enfermería, odontología y farmacia. Igual sucede con categorías cercanas como neurología, psiquiatría y neurociencias, o entre las áreas clínicas, las ciencias básicas o las categorías de salud pública (Fig. 2). Por esta razón, la agrupación de revistas de categorías diferentes con GSM en grandes áreas es inequitativa. 

**Figura 2. Producción:** de artículos y citas según categorías del Scimago Journal Ranking en el año 2016. La variabilidad en las costumbres de citación (citas/documento), y las citas recibidas (representadas por índice H) hacen que no puedan agruparse las revistas de categorías diferentes en el área de la salud con Google Scholar Metrics

Con lo expuesto, es más fácil de entender los problemas de fondo: el escaso desarrollo editorial de las revistas y el desconocimiento de Colciencias para asumir una medición de las revistas con los índices universales. Usar una sola métrica para las revistas nacionales y las internacionales homologadas, sumado a la ausencia de estrategias formativas y de apoyo a las revistas llevará a la fragilidad a la mayoría de las revistas colombianas. La competencia regional de Brasil implementó con SciELO el poderoso gestor de contenidos editoriales Scholar One^TM^ para sus revistas, mejorando los procesos editoriales y permitiendo una auditoria externa en tiempo real desde SciELO ^6^^,^^7^. Para una revista de salud latinoamericana, es más importante la indexación en PubMed que en las bases citacionales; 90 lo han logrado, entre ellas, 51 brasileñas y 5 colombianas. Pero, para Brasil es más importante la inclusión en PubMed Central, 20 han sido admitidas en los últimos cinco años, y una sola en el resto de Latinoamerica: Colombia Médica. Las citaciones y los rankings llegan con el progreso de la revista. Si no se comprende la singularidad de cada área, no es posible aplicar diferencias en las dinámicas y la forma de medir el sistema de revistas nacional.

 Colombia es el tercer país en número de revistas científicas en Latinoamérica, por lo cual es un referente regional en sus modelos de producción, edición, medición y proyección editorial. Publindex debería revaluar el sistema de medición que propuso para las próximas convocatorias, definiendo las siguientes características: mantener un sistema mixto, pero no superpuesto; con apuestas hacia el desarrollo editorial diferencial por áreas y la internacionalización; que establezca pautas y subsidie soluciones que mejoren las deficiencias organizativas, editoriales y bibliométricas de todo el sistema; pero, que desestimule el confort de las revistas que se encuentran en las clasificaciones de los cuartiles inferiores. Es necesaria la integración de las políticas de los otros sistemas de información de Colciencias, como la estimación de productos en las clasificaciones de grupos GrupLAC fortalecerán a las revistas científicas a mediano plazo. Pero, falta la promoción de la evaluación por pares que está ausente como producto en la categorización de investigadores en el CvLAC y los editores padecemos el silencio no cuantificado de los investigadores mejor calificados por Colciencias cuando son invitados a evaluar el manuscrito de un colega. Si las políticas, estrategias y convocatorias de Colciencias no incluyen y visibilizan valores como la equidad, la solidaridad y la confiabilidad con el tiempo será el máximo responsable del retraso de la investigación en el país.
